# Efficient generation of long-distance conditional alleles using recombineering and a dual selection strategy in replicate plates

**DOI:** 10.1186/1472-6750-9-69

**Published:** 2009-07-28

**Authors:** David Voehringer, Davina Wu, Hong-Erh Liang, Richard M Locksley

**Affiliations:** 1 Howard Hughes Medical Institute, Department of Medicine, University of California San Francisco, San Francisco, CA 94143-0795, USA; 2 Institute for Immunology, University of Munich, Goethestrasse 31, 80336 Munich, Germany

## Abstract

**Background:**

Conditional knockout mice are a useful tool to study the function of gene products in a tissue-specific or inducible manner. Classical approaches to generate targeting vectors for conditional alleles are often limited by the availability of suitable restriction sites. Furthermore, plasmid-based targeting vectors can only cover a few kB of DNA which precludes the generation of targeting vectors where the two *lox*P sites are placed far apart. These limitations have been overcome in the recent past by using homologous recombination of bacterial artificial chromosomes (BACs) in *Escherichia coli *to produce large targeting vector containing two different *lox*P-flanked selection cassettes so that a single targeting event is sufficient to introduce *lox*P-sites a great distances into the mouse genome. However, the final targeted allele should be free of selection cassettes and screening for correct removal of selection cassettes can be a laborious task. Therefore, we developed a new strategy to rapidly identify ES cells containing the desired allele.

**Results:**

Using BAC recombineering we generated a single targeting vector which contained two different selection cassettes that were flanked by *lox*P-*lox*P sites or by FRT-FRT/*lox*P sites so that they could be deleted sequentially by Cre- and FLPe-recombinases, respectively. Transfected ES cells were first selected in the presence of both antibiotics *in vitro *before correctly targeted clones were identified by Southern blot. After transfection of a Cre recombinase expression plasmid ES cell clones were selected on replicate plates to identify those clones which maintained the FRT-FRT/*lox*P flanked cassette and lost the *lox*P-*lox*P flanked cassette. Using this strategy facilitated the identification of ES cell clones containing the desired allele before blastocyst injection.

**Conclusion:**

The strategy of ES cell cultures in replicate plates proved to be very efficient in identifying ES cells that had undergone the correct recombination event. This approach facilitates the generation of conditional knock-out mice when large parts of the genome are intended to be flanked by *lox*P sites.

## Background

Conditional knockout mice are typically generated by insertion of two *lox*P sites flanking an exon that is critical for gene function. Crossing these mice to mice that express the Cre recombinase in a tissue-specific or inducible manner leads to deletion of the *lox*P-flanked DNA segment [1,2]. The classical approach of genetic engineering to generate targeting vectors for conditional alleles can be quite laborious since homology arms and *lox*P-flanked selection cassettes need to be combined using suitable restriction sites. The development of phage-based recombineering systems which utilize homologous recombination in *E. coli *have facilitated the generation of suitable targeting vectors (reviewed in [3]). In addition, the recombineering technology allows the generation of large targeting vectors with the possibility to introduce *lox*P sites that are separated by several kB of genomic sequence by a single targeting event in ES cells [1]. Here, we modified previously described tools to generate *il-4/il-13 *double knockout mice. We used a new strategy which facilitates the identification of correctly targeted conditional alleles by culturing ES cells in replicate plates after Cre-mediated deletion of a *lox*P-flanked resistance cassette.

## Results and discussion

We generated conditional *il-4/il-13 *double knockout mice with a single targeting vector that introduced two *lox*P sites separated by 21 kB of genomic sequence. We used two different selection cassettes with dual pro- and eukaryotic promoters that allowed selection in *E. coli *and subsequently in ES cells.

First, we retrieved a 27.6 kB segment of genomic sequence from an *il-4*/*il-13 *containing BAC clone into the low copy plasmid pBR322 by gap repair recombineering [4]. Fragments of up to 80 kB can be subcloned into pBR322 [5]. At least three reasons argue for subcloning genomic segments from BAC clones. Firstly, the retrieved genomic segments are easier to handle than BACs which grow at only one copy per cell. Secondly, BAC backbones contain a *lox*P site which should be removed from the targeting vector. Finally, identification of correct targeting events by Southern blot analysis requires that the homology arms flanking the selection cassettes are well defined and not too large.

**Figure 1:**
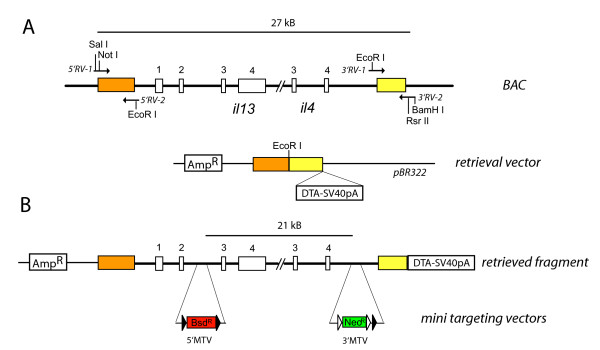
**Generation of the targeting vector**. (**A**) The retrieval vector was constructed by cloning Sal I/EcoR I and EcoR I/BamH I digested homology arms (colored boxes; about 500 bp) that had been generated by PCR amplification of indicated segments from BAC DNA into BamH I/Sal I digested pBR322. The DTA-SV40pA cassette was inserted into the Rsr II site of 3'RV-2. All four exons (open boxes) of *il-13 *and the last two exons of *il-4 *are shown. (**B**) The EcoR I-linearized retrieval vector was used to retrieve a 27 kB segment from the BAC by gap-repair in DY380 cells. The final targeting vector was generated by insertion of two mini targeting vectors containing either a blasticidin resistance cassette (5'MTV) or a neomycin/kanamycin resistance cassette (3'MTV) flanked by 350–500 bp of homology arms into the second intron of *il-13 *and downstream of *il-4 *by recombineering in DY380 cells.

The retrieval vector was constructed by cloning PCR products of about 500 bp which were homologous to sequences upstream of *il-13 *and downstream of *il-4*, respectively, into pBR322 followed by the insertion of a diphtheria toxin A (DTA) expression cassette to enable counter selection against random integration in ES cells. Although DTA could be problematic due to early extra-chromosomal expression before integration of the targeting vector, it has been used successfully in the past [6,7]. The *il-4/il-13 *encoding BAC clone was transfected into DY380 cells, a previously described cell line which was engineered to contain a heat-inducible defective λ prophage encoding the *Red *genes that are required for homologous recombination and gap-repair [5,3]. After the *Red *genes had been induced by temperature shift the cells were transformed with the linearized retrieval vector and selected on LB-Amp plates. Next, two mini-targeting vectors (MTVs) were generated to introduce *lox*P sites into the retrieved DNA segment. The MTVs were constructed in pBluescript by one-step 4-piece ligation of two 350–500 bp homology arms together with either a *lox*P-flanked blasticidin resistance (Bsd^R^) cassette (5'MTV) which was generated by exchange of the neomycin/kanamycin resistance cassette (Neo^R^) in the plasmid PL452 [4] or a FRT-FRT/*lox*P flanked Neo^R ^cassette (3'MTV) derived from plasmid PL451 [4]. The homology arms were designed to target the second intron of *il-13 *(5'MTV) or a segment downstream of *il-4 *outside known regulatory sequences (3'MTV), respectively. The targeting sites of both MTVs are separated by 21 kB of genomic sequence. Heat-induced DY380 cells containing the retrieved DNA segment were transformed sequentially with MTVs which had been excised from the pBluescript vector backbone to generate the final targeting vector (Figure 1). Transformed DY380 cells were grown on kanamycin and blasticidin containing LB plates and correct integration of the MTVs was determined by PCR. One clone was picked and expanded in 200 ml liquid culture from which the targeting vector was isolated, cut with Not I and purified using phenol/chloroform extraction. In the next step, we transfected 30 μg of the linearized targeting vector into ES cells which were then grown in medium containing G418 and blasticidin. 240 ES cell clones were analyzed by Southern blot to determine correct integration into the genome. 22 clones (9.2%) showed homologous recombination of both selection cassettes. 7 clones (2.9%) had only integrated the 3' targeting site and 2 clones (0.8%) had only integrated the 5' targeting site. This demonstrates that the frequency of correct targeting is relatively high (9.2%). However, it also indicates that the long distance between the targeting sites can lead to loss and unspecific integration of one or the other selection cassette in a significant number of clones. Comparable targeting frequencies (3 of 47 clones, 6.4%) were reported for a shaved BAC clone which contained hygromycin and neomycin resistance cassettes separated by 43 kB [1].

**Figure 2:**
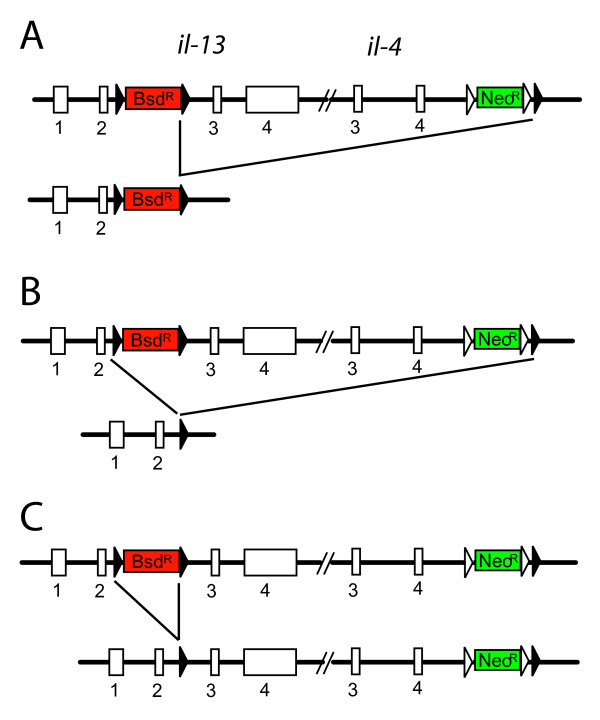
**Three possible recombination events during Cre-mediated deletion of the Bsd^R ^cassette**. Expression of Cre recombinase in targeted ES cells can lead to deletion of the Neo^R ^cassette (**A**), the Neo^R ^and the Bsd^R ^cassette (**B**) or the Bsd^R ^cassette (**C**). Closed triangles indicate *lox*P sites, open triangles indicate FRT sites.

In the next step we removed the Bsd^R ^cassette by transfection of ES cells with a Cre-recombinase encoding expression plasmid. Since 3 loxP sites are still present in the targeted allele it is possible that 3 different recombination events occur (Figure 2). The desired recombination event should only remove the Bsd^R ^cassette (Figure 2C) whereas both other recombinations would also delete the Neo^R ^cassette. We could therefore distinguish between correctly and incorrectly recombined alleles by culturing ES cells in replicate wells containing either G418 alone or G418 plus blasticidin. Cells that survived in G418 but died in G418 plus blasticidin were expected to have undergone the correct recombination event. One ES cell clone that had been targeted correctly during the first round of selection was transfected with a Cre expression plasmid and grown for 7 days in G418. Then, 120 colonies were picked and further cultured in replicate wells containing either G418 or both G418 and blasticidin. 8 subclones (6.7%) did not grow under blasticidin selection indicating loss of the Bsd^R ^cassette. Southern blot analysis of subclones revealed that all 8 subclones showed the correct recombination. Therefore, the strategy of using two different selection cassettes and culturing ES cells in replicate wells after Cre-mediated deletion of one selection cassette proved to be highly efficient and reduces the number of clones that need to be re-screened by Southern blot. An alternative strategy for selection-based identification of correctly removed resistance cassettes would be to use a *lox*P-flanked positive/negative selection cassette (e.g. Hyg^R^/tk) instead of the *lox*P-flanked Bsd^R ^cassette. Culturing ES cells in ganciclovir-containing medium after Cre-transfection would then delete all subclones that retained the Hyg^R^/tk cassette. However, this selection strategy was often inefficient in the past due to low expression of thymidin-kinase which resulted in many false-positive subclones that survived the selection [8]. In addition, adverse effect of ganciclovir on ES cell cultures have been described [9].

**Figure 3:**
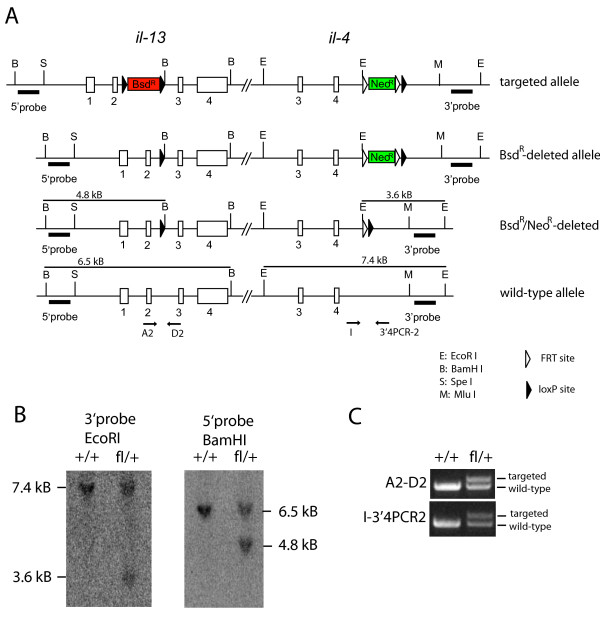
**Three steps to generate the final targeted allele**. (**A**) Illustration of the targeted allele before and after deletion of the selection cassettes. The location of the Southern probes and the restriction sites used is indicated. Filled triangles indicate *lox*P sites and open triangles indicate FRT sites. (**B**) Southern blot of tail DNA from wild-type (+/+) or heterozygous mice (fl/+) digested with BamH I (to detect the 5' targeting event) or EcoR I (to detect the 3' targeting event). (**C**) PCR of tail DNA from wild-type (+/+) or heterozygous mice (fl/+) using primers A2-D2 or I-3'4PCR-2. The larger PCR products indicate the presence of the remaining *lox*P site (A2-D2 PCR) or the FRT/*lox*P site (I-3'4PCR-2 PCR).

**Figure 4:**
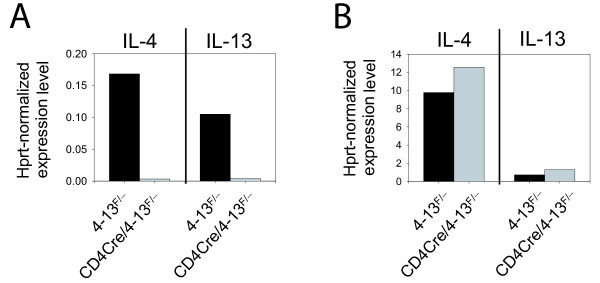
**The targeted allele is functional and can be efficiently and specifically deleted by the Cre recombinase**. RT-PCR analysis of IL-4 and IL-13 expression from Th2 polarized cells (**A**) or mast cells (**B**). Cells from 4–13^F/- ^mice (black bars) and CD4-Cre/4–13^F/- ^mice (grey bars) were analyzed. Expression levels are normalized to hprt.

ES cells were injected into C57BL/6 blastocysts to generate chimeras. After germline transmission of the targeted allele had been confirmed by PCR, we crossed the mice to FLPe deleter mice to delete the Neo^R ^cassette [10]. The final targeted allele (4–13^F^) contains a single *lox*P site in the second intron of *il-13 *and a FRT/*lox*P site downstream of *il-4 *(Figure 3). To determine the efficiency and specificity of Cre-mediated deletion of the 21 kB intergenic region, we crossed 4–13^F/+ ^mice to CD4-Cre mice [11] on IL-4/IL-13 knockout background (CD4-Cre/4-13^-/-^) [12]. RT-PCR analysis of Th2-polarized CD4 T cells or bone marrow derived mast cells of 4–13^F/- ^or CD4-Cre/4–13^F/- ^mice revealed that the targeted allele was efficiently recombined in CD4 T cells (Figure 4A) but not in mast cells (Figure 4B). The immune response of conditional IL-4/IL-13 knockout mice upon infection with helminth parasites will be described elsewhere (Sullivan *et al., submitted*). The recombination occurred with a remarkably high efficiency despite the relatively long distance between both *lox*P sites (21 kB). High recombination efficiencies are obviously essential for conditional knock-out alleles. As one might expect, the efficiency generally drops with the distance between the two *lox*P sites. However, a segment of 4 Mb on mouse chromosome 11 could still be recombined with an efficiency of 11% [13]. For even larger segments it has been shown that the logarithm of the recombination efficiency is inversely proportional to the distance between the *lox*P sites [13]. Furthermore, inter-chromosomal recombinations have been generated using the Cre/*lox*P system although the efficiency was very low (10^-5 ^to 10^-7^) [14-16].

## Conclusion

We describe a new strategy to facilitate the identification of ES cells with correctly recombined conditional alleles. This strategy is based on the use of two different *lox*P-*lox*P or FRT-FRT/*lox*P flanked selection cassettes so that ES cells could be cultured in replicate plates after Cre-mediated removal of the first selection cassette. Southern blot analysis of ES cells that remained resistant to one but lost resistance to the other antibiotic confirmed the correct recombination event. Therefore, this strategy appears very useful for rapid identification of ES cell clones containing the desired allele before blastocyst injection.

## Methods

### Generation of the targeting vector

The retrieval vector was constructed by cloning two short homologous arms, generated by PCR amplification of CT7-111I18 BAC DNA (from CitbCJ7 mouse BAC library; 129Sv origin) with the following primer sets: 5'RV-1: gtcgacgcggccgcactagtttgtgtatattg and 5'RV-2: gaattcctagaactctgtagatcag for the 5' homolgous arm and 3'RV-1: gaattcacaccaacactgacatc and 3'RV-2: ggatccggaccgcccttaatccacgcg for the 3'homologous arm. These arms were first cloned into pCR2.1-TOPO and sequenced, then cut with Sal I/EcoR I (5'RV) or BamH I/EcoR I (3'RV) and subcloned into BamH I/Sal I digested pBR322 which had been cut with EcoR I and Hind III, blunted and religated to destroy the EcoR I site. A full length diphtheria toxin alpha counter-selection cassette was then cloned by RsrII digest from pKO SelectDT (Lexicon Genetics, TX) into the Rsr II site present in primer 3'RV-2 to generate the final retrieval vector (Figure 1A). The *E. coli *strain DY380 [5] was transformed with CT7-111I18 BAC and the linearized retrieval vector according to the protocol described previously [4]. In brief, BAC-transformed DY380 cells were heat-induced by incubation at 42°C for 15 minutes, chilled on ice for 5 minutes, washed in ice-cold water and electroporated with 10 ng EcoR I linearized retrieval vector using a Bio-Rad electroporator set to 1.75 kV, 25 μF, 200 Ω. Recombinant clones were selected at 30°C on LB-Amp plates.

MTVs were constructed by PCR amplification of CT7-111I18 DNA using the following primer pairs: for the 5'MTV: primers A: 5'-tggcggccgctattggctgttggcttc-3' and B2: 5'-gagaattctgtagttaagggtcac-3'; and primers C: 5'-gaggatccttcctcatgctgtggtg-3' and D: 5'-gagtcgacgaaggccgttgaactg-3'. For the 3'MTV: primers E: 5'-aagcggccgctaacacagtagaactac-3' and F: 5'-ggaattcatgtcttgatctgaaag-3'; and primers G: 5'-gaagatctgacacagtgcctctg-3' and H: 5'-ggagtcgactggctggcctggagctc-3'. To complete the 5'MTV which was designed to target the second intron of *il-13*, PCR products AB2 and CD were cut with Not I/EcoR I and BamH I/Sal I, respectively, and cloned together with a EcoR I/BamH I digested loxP flanked blasticidin-resistance cassette (Bsd^R^) into Not I/Sal I digested pBluescript. The Bsd^R ^cassette was generated by exchange of the original neomycin/kanamycin resistance cassette (Neo^R^) in plasmid PL452 [4] for a Bsd^R ^gene isolated by Nco I/Bcl I digest from pCoBlast (Invitrogen). The 3'MTV was assembled by cloning PCR products EF and GH, digested with Not I/EcoR I and Bgl II/Sal I, respectively, together with EcoR I/BamH I digested FRT-FRT/loxP-flanked Neo^R ^cassette from plasmid PL451 [4] into Not I/Sal I digested pBluescript.

The 3'MTV was electroporated into heat-induced DY380 cells containing the retrieved BAC subsequence and colonies were selected on LB-kanamycin plates. After the targeting event was confirmed by PCR, cells were again heat-induced and electroporated with 5'MTV and selected on LB-kanamycin/blasticidin plates to generate the final targeting vector used for homologous recombination in ES cells.

### Targeting of ES cells and Cre-mediated deletion of the Bsd^R ^cassette

10^7 ^E14 ES cells (129/Ola background) were electroporated with 30 μg Not I-linearized targeting vector and clones were selected in G418 (0.25 mg/ml) and blasticidin (10 μg/ml) containing medium. 240 clones were picked and subjected to Southern blot analysis. Genomic DNA was digested with EcoR I, run on a 1% agarose gel, blotted, hybridized first with the 3'probe, then stripped and hybridized with the 5'probe. The probes were generated by PCR using CT7-111I18 DNA as template and primers 5'probe1: 5'-gtcacgagccagaccattcg-3' and 5'probe2: 5'-cactcatgagcccacagc-3' and primers 3'probe3: 5'-gagagaggaactctgggatag-3' and 3'probe4: 5'-gctgcagcaggactctactg-3'. The 3'probe shows a band at 7.4 kB for the wild-type allele and 5.5 kB for the targeted allele. The 5'probe shows a band at 17.5 kB for the wild-type and 10.4 kB for the targeted allele.

To delete the Bsd^R ^cassette 10^7 ^cells of an ES cell clone was electroporated with 10 μg supercoiled pMC-CreN plasmid which expresses the Cre-recombinase with a SV40-derived nuclear localization signal sequence under control of HSV-tk promoter/enhancer elements [17]. 1000 cells were seeded on a 10 cm dish and grown in medium containing only G418. 120 subclones were picked and grown in replicate plates, one only with G418 selection, the other one with G418 and blasticidin to identify clones that lost the ability to grow in the presence of blasticidin. 8 of 120 subclones had lost the ability to grow under blasticidin selection and were subjected to Southern blot analysis. Genomic DNA was digested with BamH I, run on a 1% agarose gel, blotted and hybridized with the 5'probe. The wild-type allele generated a band at 6.5 kB and the correctly recombined allele a band at 4.8 kB.

### Generation of mice and deletion of the Neo^R ^cassette

ES cells were injected into C57BL/6 blastocysts. Chimeric mice were bred to C57BL/6 mice and offspring were analyzed by PCR for germ line transmission. Mice that contained the targeted allele were bred with FLPe deleter mice (*Gt(ROSA)26Sor*^*tm1(FLP1)Dym*^; The Jackson Laboratory, Bar Harbour, ME) to delete the Neo^R ^cassette [10]. Successful deletion and correct targeting at both sites was demonstrated by Southern blot analysis after digestion of tail DNA with EcoR I and hybridization with the 3'probe which resulted in bands at 7.4 kB (wild-type allele) and 3.6 kB (Neo-deleted allele) or by BamH I digest and hybridization with the 5'probe resulting in bands at 6.5 kB (wild-type) and 4.8 kB (targeted allele) (Figure 3B).

In addition, both targeting events were confirmed by PCR using primers A2: 5'-cagcatggtatggagtgtg-3' and D2: 5'-cattgcaattggagatgttg-3' or primers I: 5'-cttgaatacttggtccaccg-3' and 3'4PCR-2: 5'-gaaacaggttctcattatgtag-3' (Figure 3C).

### Th2 cell polarization, mast cell culture and RT-PCR analysis

CD4 T cells from spleen and lymph nodes of CD4-Cre/4–13^F/- ^or 4–13^F/- ^mice were purified by negative selection using the MACS CD4 T cell isolation kit (Miltenyi Biotec, Germany) according to manufacturer's instructions and cultured for 5 days in the presence of 20 ng/ml IL-2, 20 ng/ml IL-4 and 20 μg/ml anti-IFN-γ to induce Th2 polarization. Bone marrow cells were cultured for 11 days in the presence of 3 ng/ml IL-3 and 3 ng/ml SCF to generate mast cells. RT-PCR was performed with cDNA from Th2 and mast cell cultures using the primers: IL-4fwd: 5'-agctagttgtcatcctgctc-3' and IL-4rev: 5'-tggtggctcagtactacgag-3', IL-13fwd: 5'-gcagtcctggctcttgcttg-3' and IL-13rev: 5'-tgctttgtgtagctgagcag-3', hprt-fwd: 5'-gttggatacaggccagactttgttg-3' and hprt-rev: 5'-gagggtaggctggcctataggct-3'. PCR reactions were performed with 58°C annealing temperature and 60 sec extension time at 72°C using the SYBR^® ^green Taq ReadyMix™ (Sigma) and Lightcycler PCR machine (Roche, Switzerland).

### Experiments with animals

All mice were housed in the specific pathogen-free animal facility at UCSF according to institutional guidelines. The experiments have been approved by the Institutional Animal Care and Use Committee (IACUC).

## Authors' contributions

DV designed and performed the experiment and wrote the manuscript. DW participated in cloning the targeting vector. HEL participated in ES cell culture. RML participated in writing the manuscript. All authors have seen and approved the manuscript.
